# Dental Pulp Cells Isolated from Teeth with Superficial Caries Retain an Inflammatory Phenotype and Display an Enhanced Matrix Mineralization Potential

**DOI:** 10.3389/fphys.2017.00244

**Published:** 2017-04-28

**Authors:** Hanaa Alkharobi, James Beattie, Josie Meade, Deirdre Devine, Reem El-Gendy

**Affiliations:** ^1^Division of Oral Biology, Leeds School of Dentistry, St. James University Hospital, University of LeedsLeeds, UK; ^2^Department Oral Biology, Faculty of Dentistry, King AbdulAziz UniversityJeddah, Saudi Arabia; ^3^Department of Oral Pathology, Faculty of Dentistry, Suez Canal UniversityIsmailia, Egypt

**Keywords:** dental pulp cells (DPCs), osteogenesis, odontogenesis, inflammation, regeneration

## Abstract

We have isolated dental pulp cells (DPCs) from three healthy (hDPCs) and three carious (cDPCs) donors and shown that compared to hDPCs cells isolated from superficial carious lesions show higher clonogenic potential; show an equivalent proportion of cells with putative stem cell surface markers; show enhanced matrix mineralization capability; have enhanced angiogenic marker expression and retain the inflammatory phenotype *in vitro* characteristic of superficial caries lesions *in vivo*. Our findings suggest that cDPCs may be used for further investigation of the cross talk between inflammatory, angiogenic and mineralization pathways in repair of carious pulp. In addition cells derived from carious pulps (almost always discarded) may have potential for future applications in mineralized tissue repair and regeneration.

## Introduction

Dental pulp cells (DPCs) contain a subset of stem cells DPSCs (Gronthos et al., 2000; Shi et al., 2001) which under appropriate conditions can differentiate into multiple lineages *in vitro* (Dominici et al., 2006; Huang et al., 2009; Martens et al., 2012). Although, evidence of differentiated functional tissue derived from transplanted DPSCs is limited some studies show that such cells can interact with biologically compatible scaffolds as part of successful tissue engineering strategies (El-Gendy et al., 2015). As an example of this transplantation of DPSCs into SCID mice was shown to result in formation of dentin pulp-like tissue structures (Gronthos et al., 2002). In situ DPCs are involved in dentin repair following damage by noxious stimuli (Gronthos et al., 2000, 2002; El-Gendy et al., 2015) and ease of access along with routine tooth banking make dental pulp an attractive potential source of multipotent cells for autologous regenerative therapies (Gronthos et al., 2000, 2002; Krebsbach and Robey, 2002; Gafni et al., 2004; Nakashima et al., 2004; Papaccio et al., 2006; Zhang et al., 2006; d'Aquino et al., 2008; Lin et al., 2008). In fact such cells have been used in maxillofacial reconstruction and periodontal ligament regeneration protocols (Huang et al., 2009; Lei et al., 2014; Ledesma-Martínez et al., 2015).

Inflammation in dental pulp induces mineralized tissue regeneration which is important for healing. This process is regulated locally by growth factors and other cytokines (Simon et al., 2011; Smith et al., 2012). In response to appropriate stimuli DPSCs differentiate into odontoblast-like cells and form reparative dentin. This mineralization process is accompanied by neo- vascularization (Freeman et al., 2015) and dental pulp is therefore considered an ideal source of mesenchymal stem cells (MSCs) for regeneration of vascularized hard tissue (El-Gendy et al., 2015). Although, there is a substantial literature on DPCs derived from healthy pulp much less information is available on DPCs derived from caries affected teeth especially with respect to stem cell characterization (Yamada et al., 2010) and whether such cells retain the same regenerative abilities as hDPCs (Gronthos et al., 2000, 2002; Huang et al., 2009; Suchanek et al., 2009; Yalvac et al., 2010; Rodríguez-Lozano et al., 2011; Eslaminejad et al., 2015). This study compares cDPCs isolated from teeth with superficial caries (Yamada et al., 2010) and hDPCs with respect to clonogenic potential, putative stem cell marker expression, mineralization capacity, expression of angiogenic genes (PECAM-1 and VEGFR2), and genes and proteins associated with the inflammatory process (TLR-2/4 and IL-6/8).

## Materials and methods

### Cell isolation and culture

Freshly extracted healthy and carious third molars were collected from adult patients. Teeth were obtained through Leeds Dental and Skeletal tissue bank (LDI Research Tissue Bank; 130111/AH/75), with informed patient consent. Carious lesions were chosen based on depth of decay in the dentin layer and assessment of this was made during sectioning of teeth both visually and with a WHO periodontal probe. Teeth with >2 mm of sound dentin measured from the edge of the carious lesion to the pulp tissue were included in this study and categorized as shallow caries (McLachlan et al., 2004; Bjørndal, 2008; Kim et al., 2010). DPCs were isolated by enzymatic digestion of pulp tissue as previously described (Alkharobi et al., 2016). hDPCs and cDPCs at passage 4 were seeded in 6-well plates at 10^5^ cells per well under basal conditions (α-MEM 20% FBS, 200 mM L-glutamine, and 100 U/mL Pen Strep). At approximately 80% confluency cells were cultured in triplicate under basal or matrix mineralization conditions (basal medium + 10 nM dexamethasone, 100 μM L-ascorbic acid). Cultures were routinely terminated at 1 and 3 wk. for qRT-PCR analysis cytokine assay and histochemical staining [Alkaline Phosphatase (ALP) and Alizarin red stains]. Experiments were performed with cells derived from three healthy and three carious donors with technical triplicates at each time point and for each culture condition.

### Colony forming efficiency assay

Freshly isolated cells from both hDPCs and cDPCs (*n* = 3 in both instances) were seeded at 10^6^ cells/dish in 10 cm Petri dishes and cultured under basal conditions for 14 days, washed with PBS and fixed with absolute ethanol for 20 min. Cells were stained with 10% (v/v) Trypan Blue for 5 min and washed gently with distilled water. Aggregates of >50 cells were defined as a colony and were counted under the light microscope.

### Flow cytometry

Appropriate antibody dilutions were determined by titration and isotype controls were used in conjunction which each test antibody (see Table 1). Staining was performed essentially according to manufacturer protocols. Briefly 10^6^ cells were incubated with 0.1% Fixable Viability Stain (FVS) in 1 ml PBS at 4°C for 30–60 min in the dark. Cells were washed 2 × 2 ml with staining buffer and Fc receptors were blocked (0.5% blocking solution 10 min RT). Cells were then incubated with CD146/PE-Cy7, CD90/PerCP-Cy5.5, CD105/BV421, CD45/APC-Cy7, and CD31/FITC in 100 μL of staining buffer and incubated at 4°C for 30–60 min. Brilliant stain buffer was added (50 μl) to reduce non-specific interaction between polymer based brilliant violet dyes and to improve the staining quality when multiple dyes are used in the same experiment. Following antibody incubation cells were washed and re-suspended gently in 500 μl staining buffer. Analysis was performed on an LSRII FACS analyser (BD Biosciences) using 405, 488, and 640 nm laser excitation. Data was collected and analyzed using both FACS DivA software (BD Biosciences) and FlowJoV10 (Tree Star). Single color stained CompBeads were used for purpose of compensation. Unstained cells, cells labeled with mouse IgG1 Isotype and fluorescence minus one (FMO) were also used as controls as described elsewhere (Roederer, 2001).

**Table 1:** **Dilutions of test and isotype control antibodies used in FACS analysis of hDPC and cDPC cell surface marker expression**.

**Test antibody**	**Dilutions**	**Isotype control**	**Dilutions**	**Supplier**
PE-Cy7	1:40	PE-CY7	1:80	BD Biosciences
Mouse Anti-Human		Mouse IgG1, κ		
CD146				
PerCP-Cy™ 5.5	1:20	PerCP-Cy™ 5.5	1:40	BD Biosciences
Mouse Anti-Human		Mouse IgG1, κ		
CD90				
BV421	1:5	BV421	1:6	BD Biosciences
Mouse Anti-Human		Mouse IgG1, κ		
CD105				
APC-Cy7	1:20	APC-Cy7	1:20	BD Biosciences
Mouse Antihuman		Mouse IgG1, κ		
CD45				
FITC	1:4	FITC	1:4	BD Biosciences
Mouse Anti-Human		Mouse IgG1, κ		
CD31				

### qRT-PCR

Mineralization (*ALP, RUNX-2, OC)*, angiogenic (*VEGFR2* & *PECAM1)* and inflammatory *(TLR-2, TLR-4)* marker expression was assessed using TaqMan based qRT-PCR as described previously (Alkharobi et al., 2016). *GAPDH* was used as housekeeping gene. Basal expression of markers is expressed as 2^−ΔCt^ relative to GAPDH and changes in gene expression under differentiating v basal conditions were determined using the 2^−ΔΔCt^ method also as described previously (Alkharobi et al., 2016) Data are presented as mean ± SD (*n* = 3) and represent triplicate technical repeats from each of 3 healthy and 3 caries derived DPC cultures.

### Cytokine assay

BD™ CBA Human Inflammatory cytokine kit was used to quantify Interleukin-8 (IL-8), and interleukin-6 (IL-6) protein levels in media conditioned by hDPCs and cDPCs grown under basal and mineralizing conditions for 1 and 3 wk. Assays were performed exactly according to the manufacturer's protocol. Data acquisition and analysis was by flow cytometer LSR II 4 Laser using flow cytometry analysis program (FCAP) array software.

### Statistics

Differences between hDPCs and cDPCs were analyzed using 1-way analysis of variance (ANOVA) followed by Tukey's multiple comparisons test (Prism 6.0). Significance was reported at *p* < 0.05.

## Results

### Colony forming unit (CFU) efficiency

**Figure 1:**
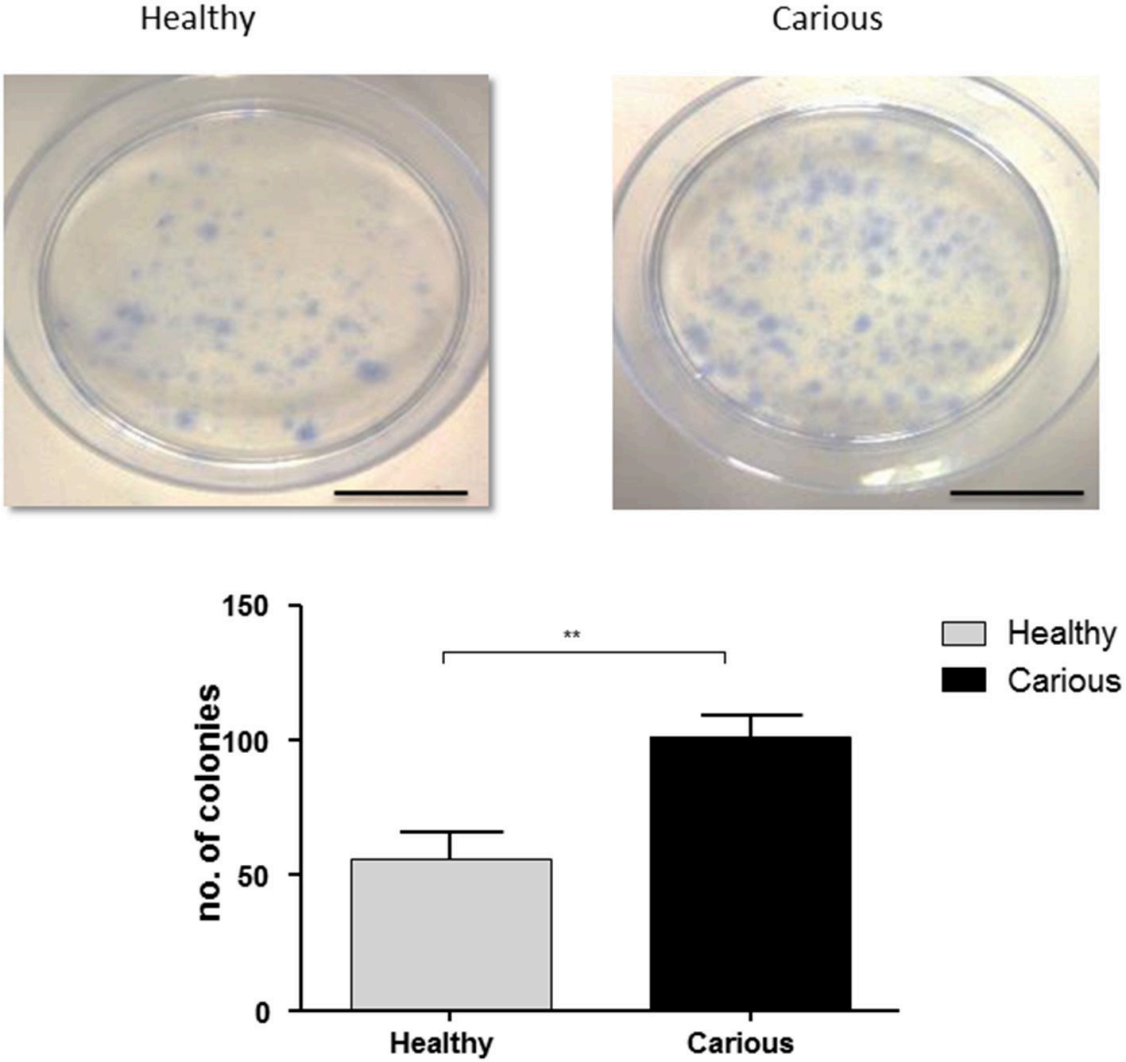
**Colony formation in hDPC and cDPC cultures**. Cells were cultured for 14 days under basal conditions and stained with Toluidine blue as described in Methods. Scale bar = 5 cm. Cultures from 3 donors for each of hDPC and cDPC were performed and a representative image is shown. Data from all cultures is shown in the lower panel and reported as mean ± SD (*n* = 3). *P* < 0.01 (Student's unpaired *t*-test).

Colonies were counted in primary basal cultures of hDPCs (*n* = 3) and cDPCs (*n* = 3) isolated from third molars. Colonies were identified as clusters of >50 cells. Both hDPCs (60 ±10) and cDPCs (100 ± 7.6) formed CFUs. A significant increase in CFUs was seen in cDPCs compared to hDPCs (*p* = 0.0053) suggesting an increased colony forming efficiency in cDPCs (Figure 1).

### Cell surface markers

**Figure 2:**
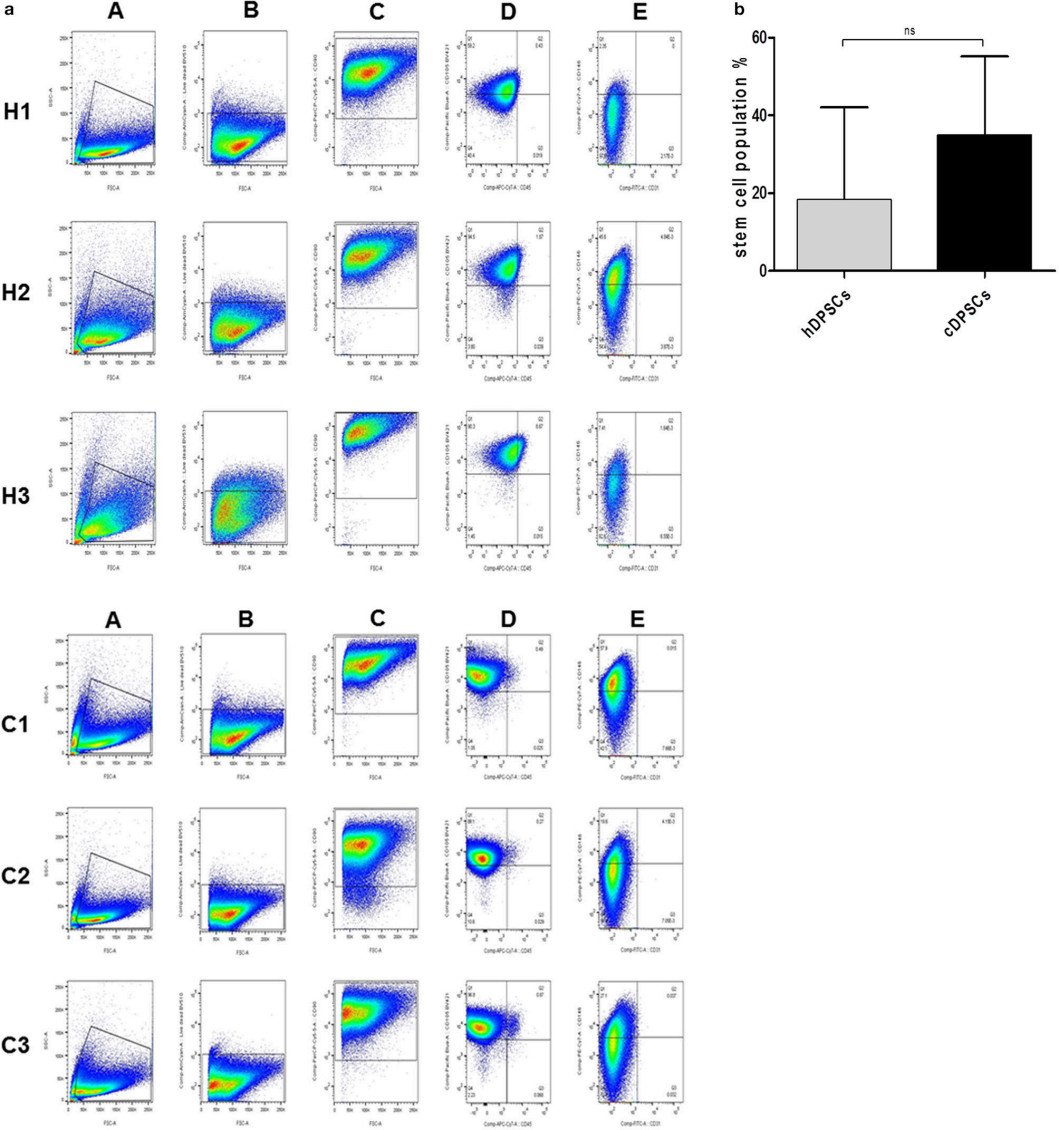
**(a)** Gating strategy identifies CD90+/CD105+/CD146+/CD31−/CD45− population in DPCs derived from three healthy (H1, H2, and H3) and three carious (C1, C2, and C3) donors. (A) Representative dot plots of intact cellular bodies gating in DPCs. (B) Representative dot plots of living cells gating in DPCs using fixable viability dye. (C) Representative dot plots of CD90+ cells gating in DPCs. (D) Representative of dot plots of CD105 (Y axis) against CD45 (X axis) surface markers from selected CD90+ subpopulation. (E) Representative dot plots of CD146 (Y axis) against CD31 (X axis) surface markers from selected CD105+/CD45− subpopulation in the previous plot. **(b)** Percentage CD90+/CD105+/CD146+/CD31−/CD45− in hDPCs and cDPCs. Data are presented as mean ± SD (*n* = 3); *p* = 0.414.

Flow cytometric analysis was used to characterize surface marker expression of cDPCs vs. hDPCs using a gating strategy to isolate the CD90+/CD105+/CD146+/CD45-/CD31- cells as a putative stem cell population (Figure 2). The data suggested that the cDPCs expressed a higher percentage stem cell population (34 ± 16.6%) compared with hDPCs (18.5 ± 19.3%) although there was a large amount of variation in the data mainly due to variation in the expression of CD146 (Table 2) such that no significant difference was apparent (see also Supplementary Figure 1).

**Table 2:** **Percentage positive cell staining for cell surface markers in h and cDPCs (see also Supplementary Figure 1)**.

**GROUP**	**Positive stem cell markers**			**Negative stem cell markers**	
	**CD146**	**CD105**	**CD90**	**CD45**	**CD31**
hDPSCs	23.2% ± 22.6	98.9% ± 1.2	97.8% ± 1.6	5.2% ± 4.94	0.04% ± 0.03
cDPSCs	43.5% ± 17.14	99.70% ± 0.36	99.60% ± 0.08	0.72% ± 0.31	0.10% ± 0.05
*P*-value	0.372	0.451	0.254	0.329	0.202

### Histochemical staining

**Figure 3:**
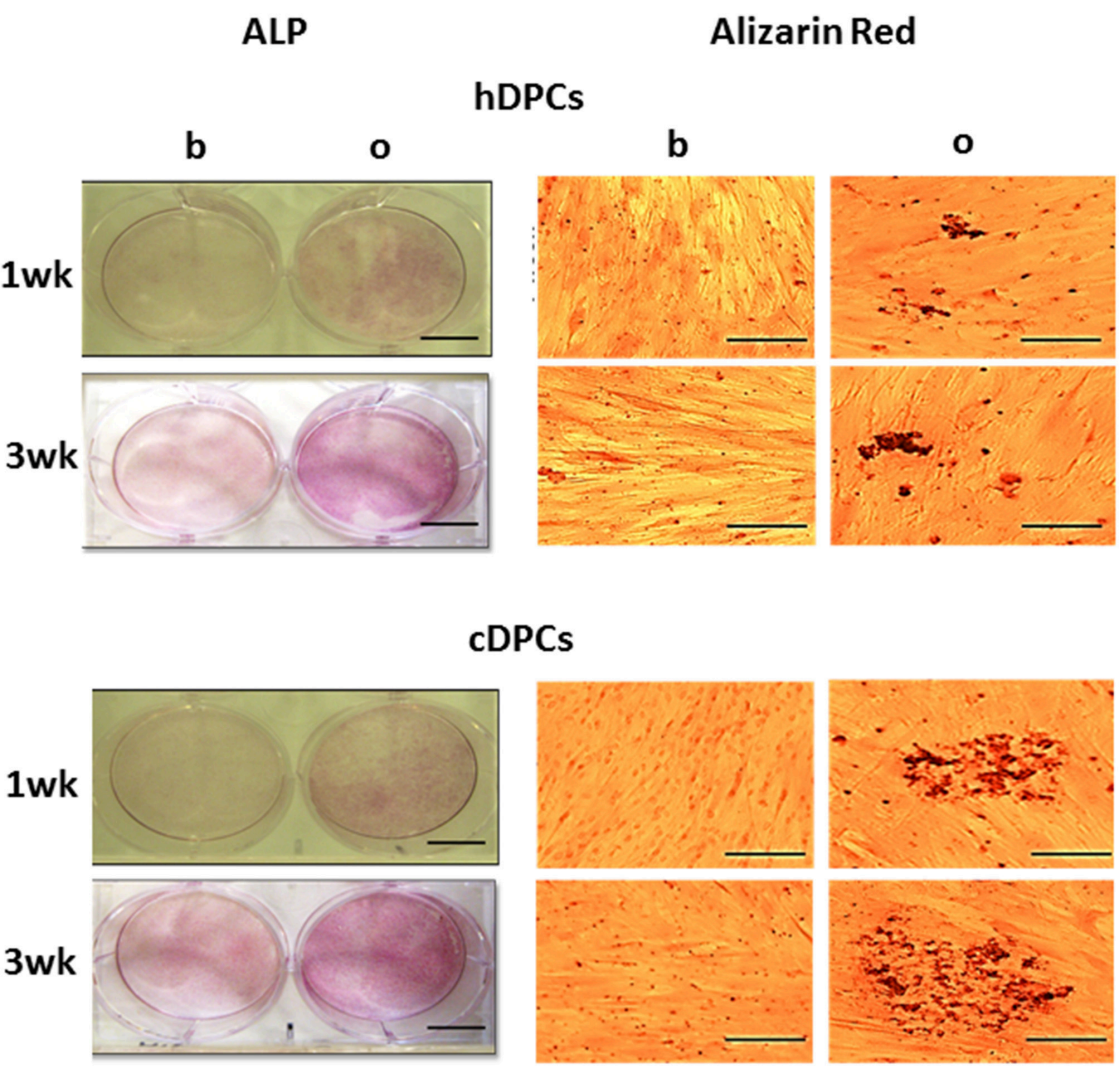
**ALP (left panels, scale bar= 5 cm) and Alizarin red (right panels, scale bar = 100 μm) staining of hDPCs (upper panels) and cDPCs (lower panels) cultured under basal (b) or mineralizing (o) conditions for 1 and 3 wk**.

Positive ALP staining was seen in cultures from all donors in both hDPCs and cDPCs groups under basal and mineralizing conditions at 1 and 3 wk. (Figure 3) However, the results clearly indicated more intense staining under mineralizing conditions at both 1 and 3 wk. There was evidence that cDPCs showed increased ALP staining under mineralizing conditions at 1 and 3 wk. compared with hDPCs. One donor from each group is presented in Figure 3 and similar results were obtained using DPCs from other donors. Matrix mineralization (as determined by Alizarin Red staining) was enhanced under differentiation conditions at both 1 and 3 wk. time points for both hDPCs and cDPCs cultures. As for ALP staining there was evidence for increased Alizarin Red staining in cDPCs compared to hDPCs (Figure 3).

### Gene expression: basal conditions

**Figure 4:**
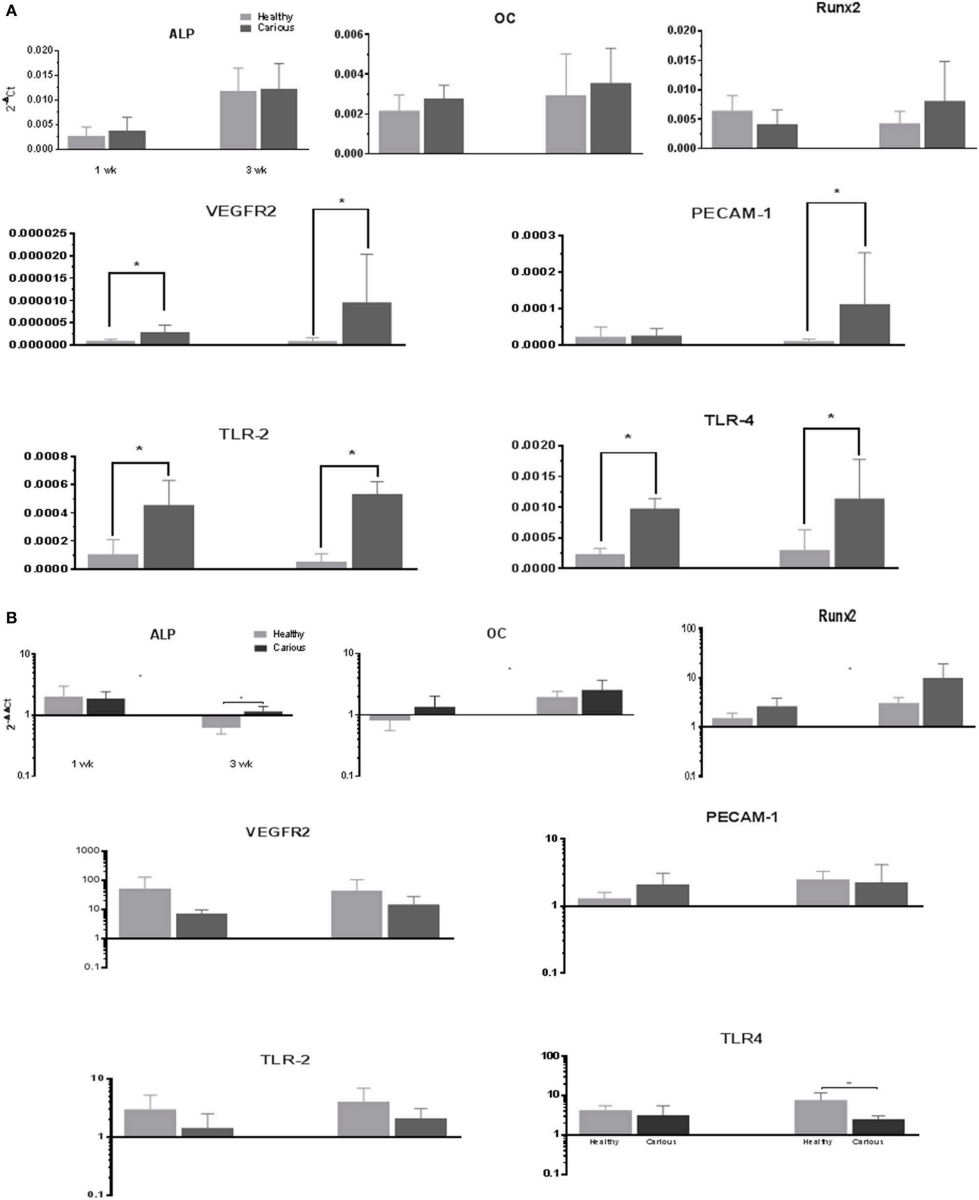
**(A)** qRT-PCR analysis of basal expression of mineralization, angiogenic and inflammatory markers in hDPCs and cDPCs. Data is presented for both 1 and 3 wk time points expressed relative to GAPDH (2^−ΔCt^) and represents triplicate technical replicates for each of 3 healthy and 3 carious donors; pooled data are expressed as mean ± SD (*n* = 3) ^*^p < 0.05. **(B)** Changes in marker gene expression under mineralizing conditions. Data is presented for both 1 and 3 wk time points expressed as fold change (2^−ΔΔCt^) mineralizing v basal and represent triplicate technical replicates for each of 3 healthy and 3 carious donors; pooled data are expressed mean ± SD (*n* = 3) ^*^p < 0.05

The expression of mineralization (*ALPL, OC*, and *RUNX-2)*, angiogenic (*VEGFR-2, PECAM-1*) and inflammatory (*TLR-2, TLR-4*) markers, was investigated in primary cultures of hDPCs and cDPCs grown under basal conditions (Figure 4A). *ALP* expression was significantly increased in hDPCs and cDPCs at wk.3 compared with wk.1 under basal conditions. *OC* and *RunX2* were expressed at approximately the same level as *ALP* although there was no time dependent change in expression of these genes. The angiogenic markers *VEGFR-2* and *PECAM1* were expressed at lower levels than mineralization markers (compare y-axis scales) approximately 1,000-fold for *VEGFR2* and 10-fold for *PECAM-1*. At the 3wk time point there was a trend for higher expression of both *VEGFR2* and *PECAM-1* in cDPCs and although this reached statistical significance for *VEGFR2*, data was characterized by high SD values. The inflammatory markers *TLR-2* and *TLR-4* were expressed at 10-fold lower levels than mineralization markers in both hDPCs and cDPCs. However the most striking feature was that both inflammatory markers were upregulated in cDPCs when compared with hDPCs (approximately 4- to 5-fold) at both 1 and 3wk time points.

### Gene expression: mineralizing conditions

Changes in the gene expression in primary cultures of hDPCs and cDPCs grown under mineralizing conditions were investigated (Figure 4B). *ALP* expression was upregulated in both cell types at wk1 and downregulated in hDPCs at wk3. *OC* expression was upregulated at wk3 in both hDPCs and cDPCs. *RUNX-2* expression was increased at both 1 and 3 wk in both hDPCs and cDPCs although there was a trend for greater upregulation at wk 3 compared to wk 1. The angiogenic markers *VEGFR-2 and PECAM-1* were also consistently upregulated under mineralizing conditions in both cell types compared to basal controls. *VEGFR-2* expression was lower in cDPCs compared to hDPCs under mineralizing conditions at 1 and 3 wk. No statistical difference in the level of upregulation for hDPC v cDPC was apparent *TLR-2* expression was upregulated under mineralizing conditions in both hDPCs and cDPCs although this effect was more apparent at the 3 wk time point. *TLR-4* expression was also upregulated under mineralizing conditions in both cell types and at both time points compared to basal controls. At wk 3 cDPCs showed significantly lower levels of *TLR-4* compared to hDPCs.

### Interleukin expression

**Figure 5:**
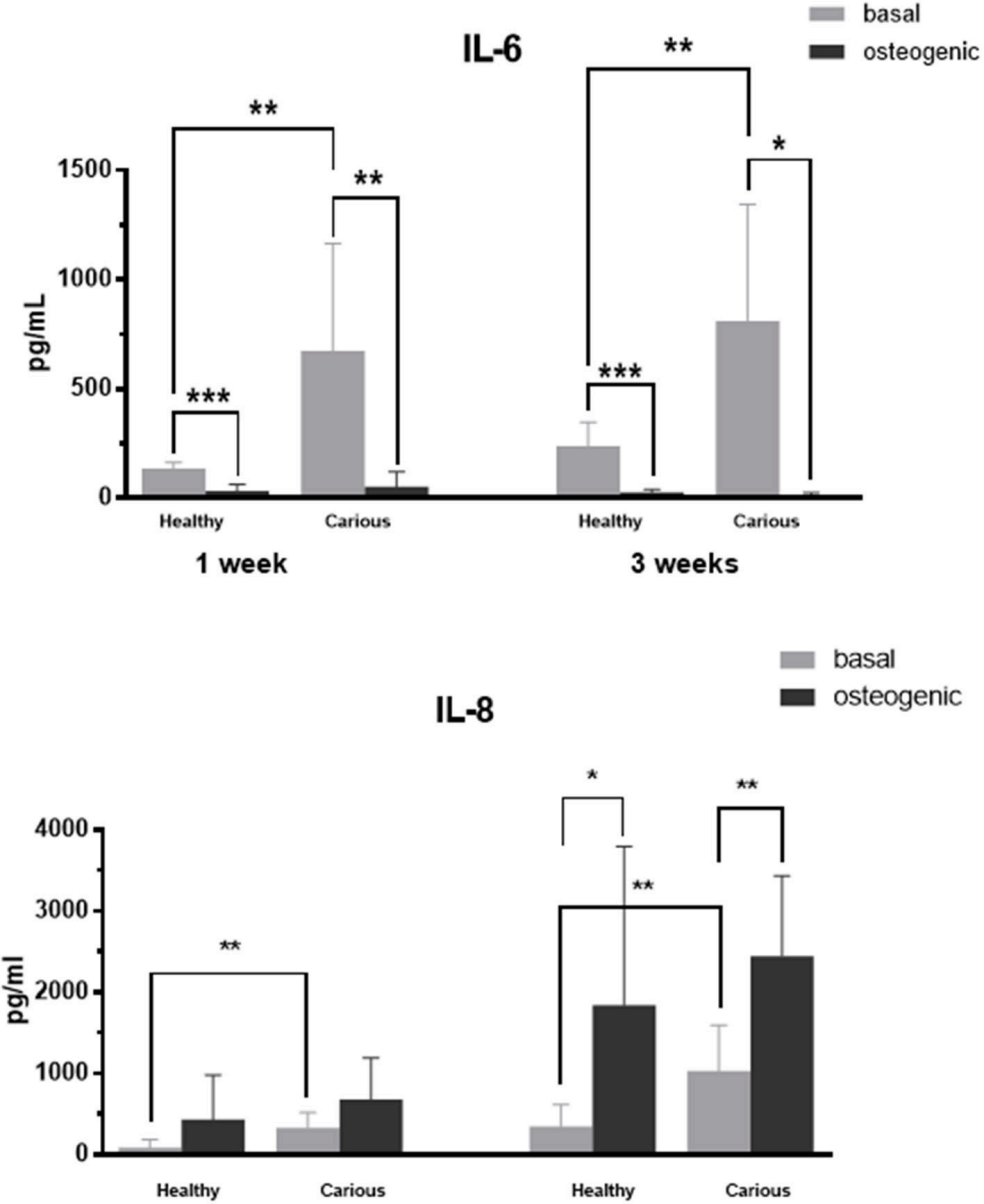
**IL-6 (upper panels) and IL-8 (lower panels) concentration in media conditioned by hDPCs and cDPCs cultured under basal and mineralizing conditions for 1 and 3 wk**. Data are shown as pg/ml and represent triplicate technical replicates for each of 3 healthy and 3 carious donors; means ± SD (*n* = 3). ^*^P < 0.05; ^**^P < 0.01; ^***^P < 0.0001.

IL-6 and IL-8 proteins were secreted by primary cultures of hDPCs and cDPCs (Figure 5). IL-6 levels were significantly lower in medium conditioned by cells grown in mineralization medium for both hDPCs and cDPCs. In addition, under basal conditions IL-6 levels were significantly higher in carious compared to healthy DPC cultures. In contrast IL-8 levels were higher in medium conditioned by cells grown under mineralizing conditions compared to cells grown under basal conditions although this was only statistically significant at 3 wk. Similarly to IL-6, under basal conditions IL-8 concentrations were higher in cDPCs compared to hDPCs at both 1 and 3 wk time points (Figure 5).

## Discussion

There is very little data on the characterization of DPCs isolated from teeth with shallow caries (cDPCs). In this report we have characterized several properties of these cells in comparison with cells isolated from healthy dental pulp tissue (hDPCs). We reasoned that some understanding of the inflammation-regeneration processes might help to determine whether the cDPCs can be used for mineralized tissue regeneration, either *in situ* for dentin/pulp complex formation or as a source of autologous stem cells for bone regeneration. We found that colonies of cDPSCs occurred at higher frequency in comparison to hDPSCs (Figure 1) in agreement with previous observations showing a higher clonogenic potential of cDPCs isolated from third molars with deep caries (Ma et al., 2012). This increased clonogenic potential of cDPCs may reflect the retention of a phenotype associated with early dentinal repair processes in response to pathological stimuli.

We confirmed that both hDPCs and cDPCs demonstrated the classical MSC surface marker profile CD146^+^/CD105^+/^CD90^+^/CD45^−^/CD31^−^ (Dominici et al., 2006; Huang et al., 2009; Martens et al., 2012; see Figure 2A). The levels of expression of CD90 and CD105 reported in the current study are largely in agreement with other studies, which reported high expression (>95%) of both CD90 and CD105 in dental pulp tissues isolated from normal healthy teeth (Lindroos et al., 2008; Eslaminejad et al., 2015). We found that CD90 and CD105 were comparably expressed in hDPCs and cDPCs. Although, previous analysis of cDPCs showed increased expression of CD90 and CD146 compared to hDPCs these studies were performed on cDPCs isolated from teeth with irreversible pulpitis or from deep carious lesions (Alongi et al., 2010; Ma et al., 2012) and this may explain the differences between these studies and our current findings. In addition previous studies typically isolated DPCs from pulp chambers only, while in the present study cells were isolated from the pulp chamber and upper portion of the root canal and this may also account for some of the observed differences. We found that CD146 was expressed in around 23% of the hDPCs. Higher levels of expression (>80%) were reported in a previous study although this may be age related as young donors (aged 16–18 year.) were used in that study (Bakopoulou et al., 2011). In addition high vascularity is needed to complete root formation at the eruption stage and CD146 is expressed in perivascular MSCs (Baksh et al., 2007). In our study hDPCs and cDPCs showed low reactivity toward the hematopoietic stem cell marker CD45 (leukocyte common antigen) and this agrees with previous data which indicated that DPCs were either negative for CD45 or expressed this marker at very low concentrations <1–2% of the cell population (Lindroos et al., 2008; Huang et al., 2009; Bansal and Jain, 2015; Isobe et al., 2015) It was reported that leukocytes represent <1% of dental pulp cell population after enzymatic digestion and harvesting (Gaudin et al., 2015) although early passages of heterogeneous stromal cell populations may still contain traces of a CD45 positive population. hDPCs and cDPCs did not express the endothelial cell marker CD31 and this also agrees broadly with other reports (Lindroos et al., 2008; Vishwanath et al., 2013).

Previous studies have shown that hDPCs can differentiate to a matrix mineralization phenotype (Gronthos et al., 2000, 2002; d'Aquino et al., 2007; El-Gendy, 2010), while controversial data have been published regarding cDPCs isolated from permanent (Alongi et al., 2010; Wang et al., 2010; Pereira et al., 2012; Ma et al., 2014; Yazid et al., 2014) or deciduous (Yu et al., 2014; Werle et al., 2015) teeth affected by deep caries. Our data clearly show that cDPCs isolated from shallow carious lesions can differentiate to this phenotype and form a mineralized matrix (Figure 3). In fact cDPCs appeared to exhibit a higher differentiation potential compared with hDPCs as assessed by mineralization assay and marker gene expression (Figure 3) although there was no statistically significant difference in mineralization marker gene expression between hDPCs and cDPCs under either basal or mineralizing conditions (Figures 4A,B respectively). Nonetheless differentiation of cDPCs may be affected by the inflammatory micro-environment and this may argue for the retention of this phenotype in cultured cDPCs. Although, this requires further experimental verification such observations are consistent with earlier studies which showed that pro-inflammatory cytokines promoted the mineralization of DPCs (Yang et al., 2012).

Under basal conditions we report the up regulation of VEGFR2 and PECAM-1 in DPCs derived from carious dental pulp compared to cells derived from healthy pulp (Figure 4A). This agrees with previous findings in inflamed dental pulp and may occur in response to the inflammatory cytokines that are released into the pulp interstitial fluid during inflammation (Cohen et al., 1996; Chu et al., 2004). We also found up regulation of the inflammatory markers TLR-2 and TLR-4 under basal conditions in cDPCs v hDPCs and some evidence suggests cross talk between the TLR and VEGF axis to co-ordinate neo-angiogenic and inflammatory responses during tissue repair at site of injury (Akira et al., 2006; Bachmann et al., 2006; Pazgier et al., 2006).

The role of inflammatory cytokines during mineralized tissue repair is also controversial. We found that IL-6 and IL-8 were secreted into media conditioned by DPCs and there was a trend for higher concentrations of IL-6 and IL-8 in media conditioned by cDPCs compared with hDPCs under basal conditions (Figure 5). These findings are supported by earlier studies, which found that IL-6 and IL-8 expression was higher in carious than in heathy pulp (Zehnder et al., 2003; McLachlan et al., 2004; Silva et al., 2009) and argues that *in vitro* cDPCs retain an inflammatory phenotype. IL-6 was down-regulated in both hDPCs and cDPCs under mineralizing conditions. However treatment with the synthetic glucocorticoid dexamethasone inhibits IL-6 expression in different cell/tissue culture systems (Malaval et al., 1998; Liu et al., 2002; Cooper et al., 2014) and dexamethasone-free induction medium is required to examine whether IL-6 expression remains down-regulated. In contrast, IL-8 secretion into conditioned medium increased under mineralizing conditions. This agrees with previous reports showing increased IL-8 expression under mineralizing conditions in human MSCs (Pereira et al., 2009) and up-regulation of IL-8 in bone marrow MSCs during BMP induced differentiation (Zachos et al., 2006).

In conclusion we have shown that, compared to healthy DPCs, cells derived from pulp with superficial caries involvement (cDPCs)—show higher clonogenic potential; have an equivalent proportion of putative stem cell populations; show enhanced matrix mineralization capability; have enhanced angiogenic marker expression; retain the inflammatory phenotype *in vitro* characteristic of superficial caries lesions *in vivo*. These findings suggest that cDPCs may be used for further investigation of the cross talk between inflammatory, angiogenic, and mineralization pathways in repair of carious pulp. In addition cells derived from carious pulps (almost always discarded) may have potential for future applications in mineralized tissue repair and regeneration.

## Author contributions

HA conducted experiments, collected and analyzed data, and contributed to writing the manuscript. RE conducted experiments, collected and analyzed data, designed experiments and contributed to writing the manuscript. JM and DD analyzed data and contributed to writing the paper. JB designed experiments and prepared the final version of the manuscript.

### Conflict of interest statement

The authors declare that the research was conducted in the absence of any commercial or financial relationships that could be construed as a potential conflict of interest.

## Supplementary material

Supplementary Figure 1 **Single parameter histograms showing the expression of cell surface markers in hDPSCs (H1, H2, H3) and cDPSCs (C1, C2, C3) cultures under basal conditions**. Data are shown for isotype controls (gray) and positively stained cells – blue (hDPCs) and red (cDPCs). **(a)** CD146 (PE-Cy7); **(b)** CD90 (PerCP-5.5); **(c)** CD105 (BV421); **(d)** CD45 (APC-Cy7); **(e)** CD31 (FITC). Percentage positively stained cells are indicated for each marker.
