# Supplementary figures and images for: Dental Pulp Cells Isolated from Teeth with Superficial Caries Retain an Inflammatory Phenotype and Display an Enhanced Matrix Mineralization Potential
Source: Front Physiol. 2017 Apr 28;8:244. doi: 10.3389/fphys.2017.00244 (PMC5408163; doi:10.3389/fphys.2017.00244)

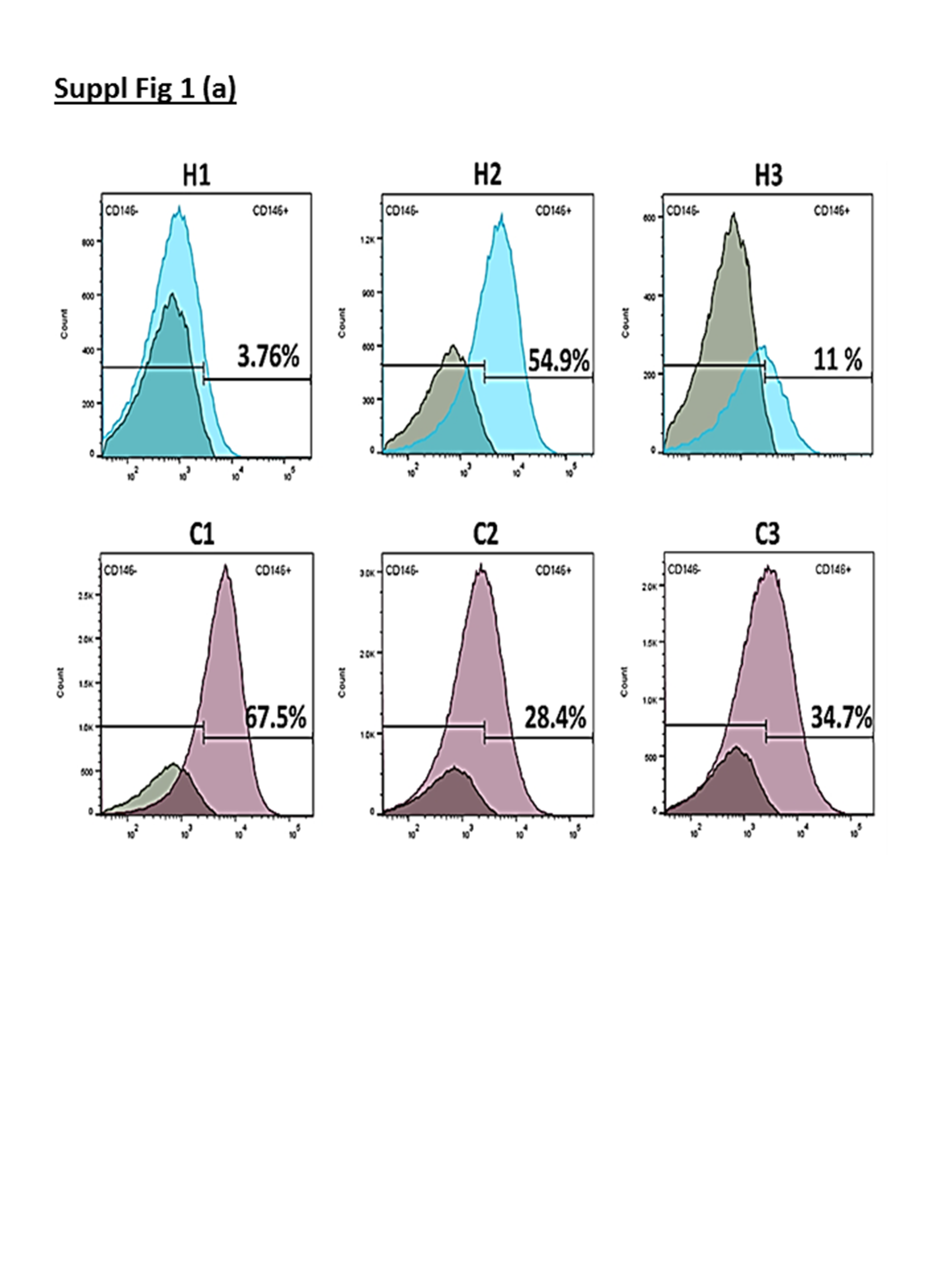

Supplement: Supplementary Figure 1 — Single parameter histograms showing the expression of cell surface markers in hDPSCs (H1, H2, H3) and cDPSCs (C1, C2, C3) cultures under basal conditions. Data are shown for isotype controls (gray) and positively stained cells – blue (hDPCs) and red (cDPCs). (a) CD146 (PE-Cy7); (b) CD90 (PerCP-5.5); (c) CD105 (BV421); (d) CD45 (APC-Cy7); (e) CD31 (FITC). Percentage positively stained cells are indicated for each marker. [file Image1.TIF]

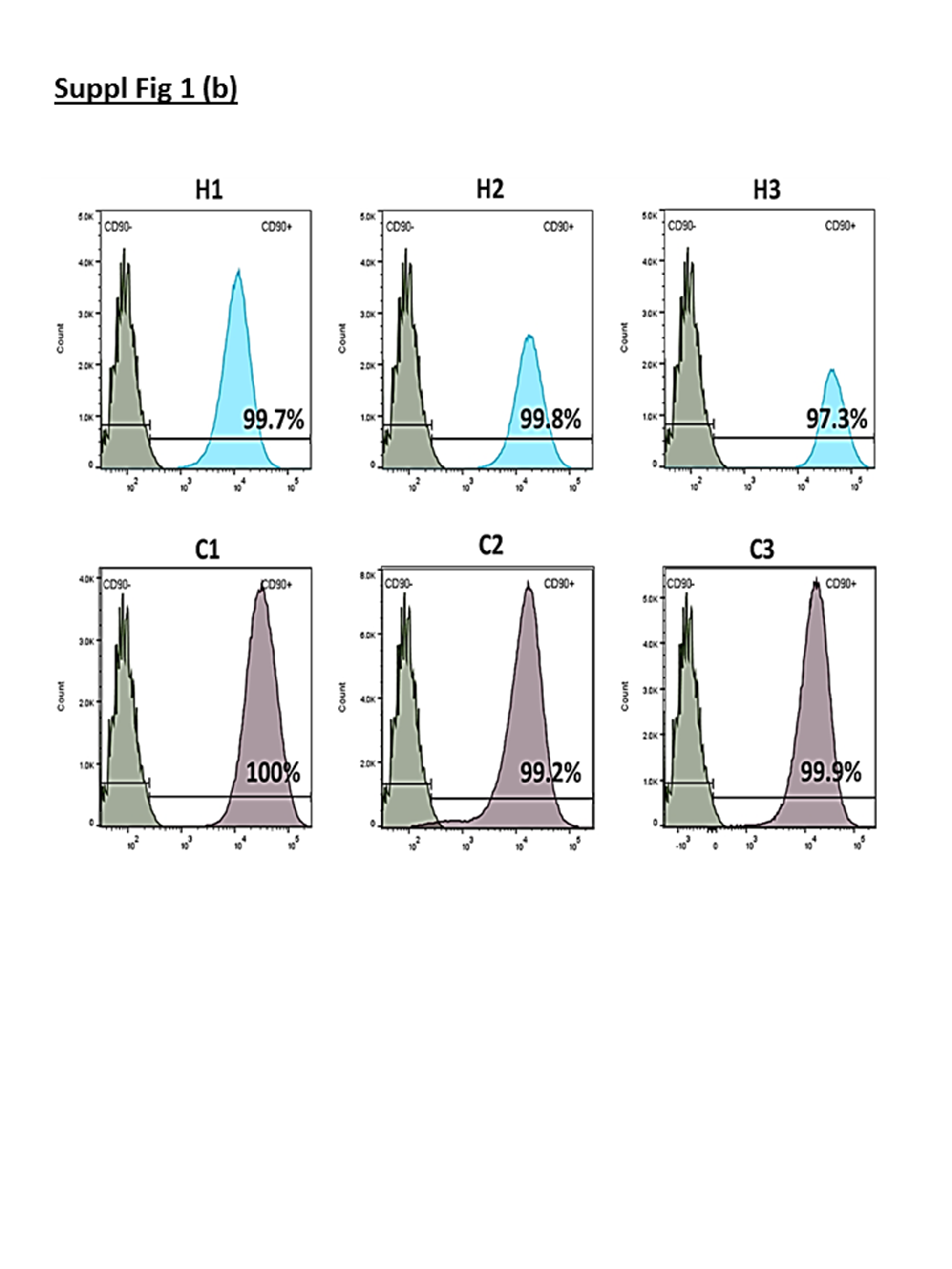

Supplement: Supplementary file 2 [file Image2.TIF]

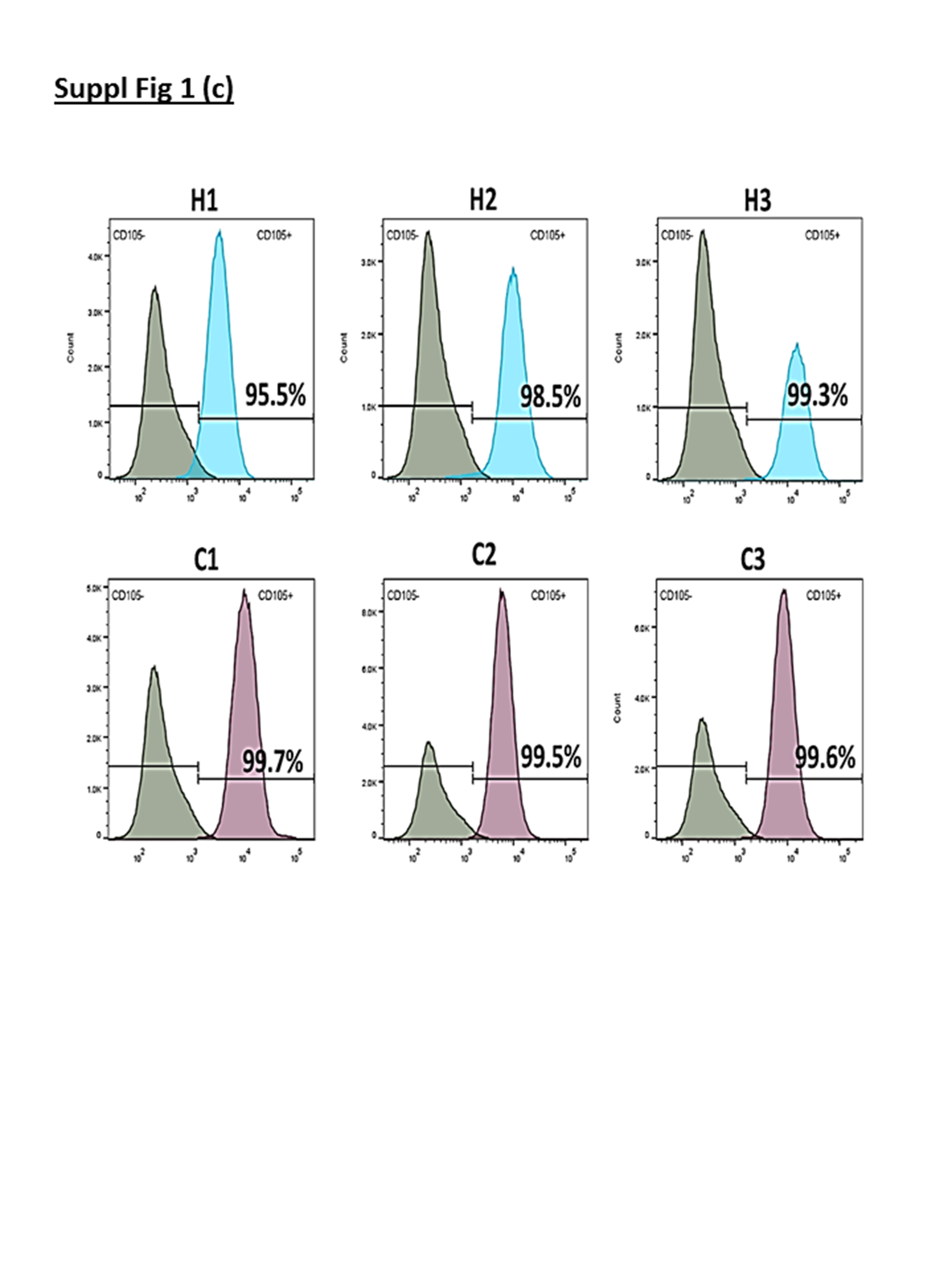

Supplement: Supplementary file 3 [file Image3.TIF]

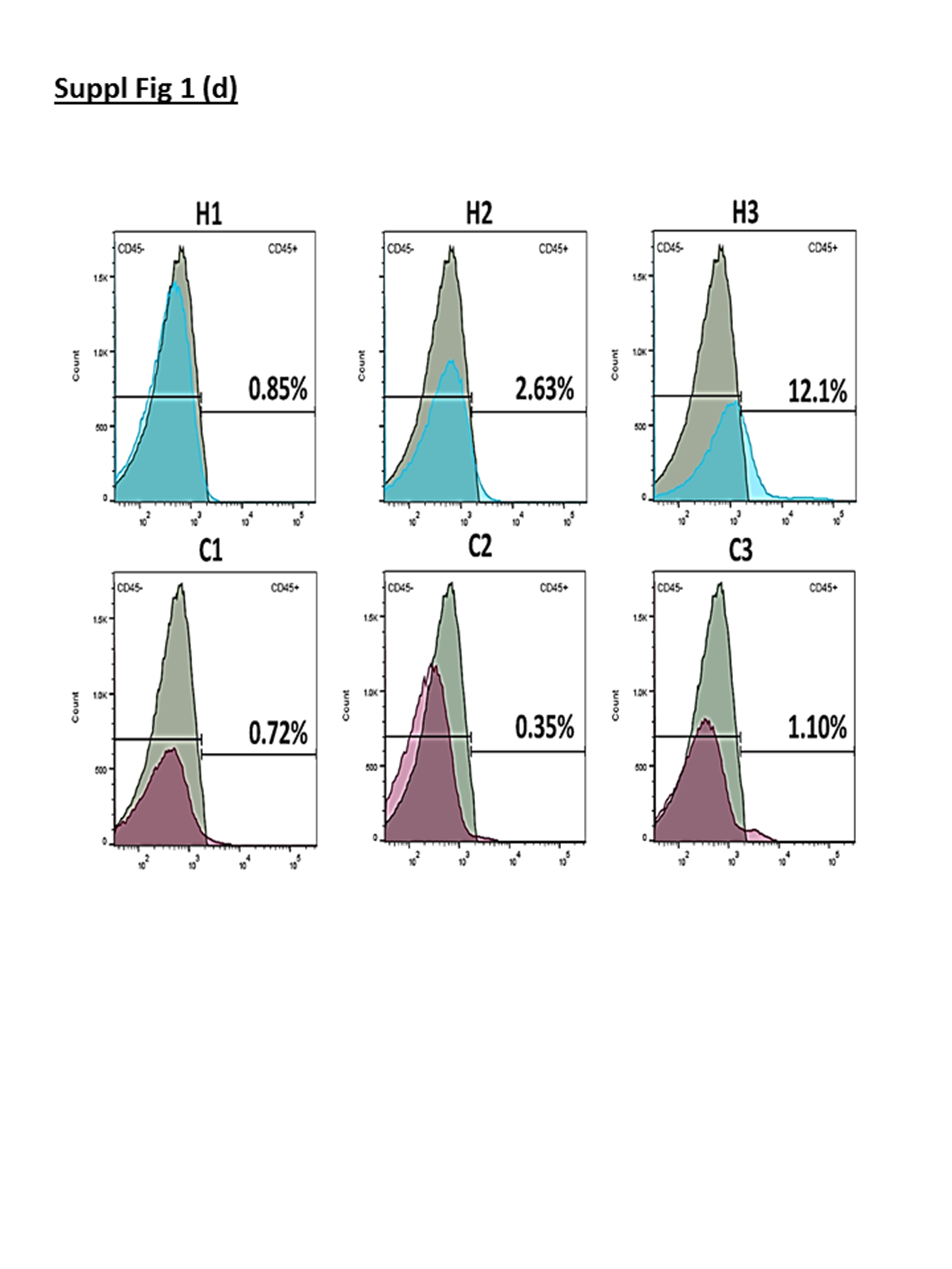

Supplement: Supplementary file 4 [file Image4.TIF]

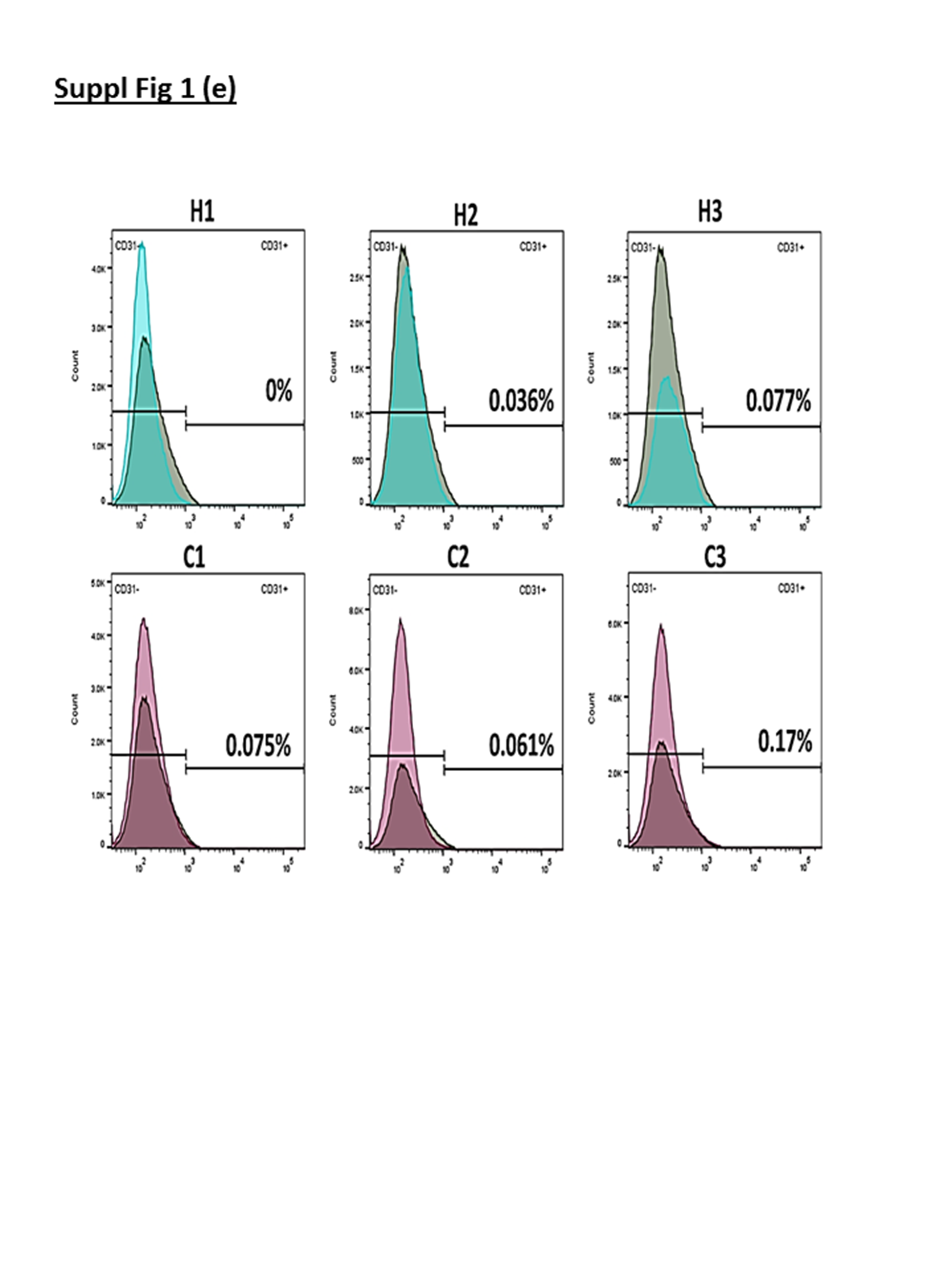

Supplement: Supplementary file 5 [file Image5.TIF]
